# Sciatic Nerve Palsy following Curved Periacetabular Osteotomy

**DOI:** 10.1155/2020/8569285

**Published:** 2020-03-19

**Authors:** Masahiro Fujita, Shinya Hayashi, Kenichi Kikuchi, Yoshinori Takashima, Tomoyuki Kamenaga, Masanori Tsubosaka, Koji Fukuda, Koji Takayama, Shingo Hashimoto, Takahiro Niikura, Ryosuke Kuroda, Tomoyuki Matsumoto

**Affiliations:** Department of Orthopedic Surgery, Kobe University Graduate School of Medicine, 7-5-1, Kusunoki-Cho, Chuo-Ku, Kobe, Hyogo 650-0017, Japan

## Abstract

Curved periacetabular osteotomy (CPO) is used for the treatment of dysplastic hips. Previous studies have reported satisfying outcomes and low rate of severe complications associated with this procedure; however, no case of postoperative sciatic nerve palsy has been reported. In this study, we describe a case of postoperative sciatic nerve palsy following CPO due to nerve strangulation by scar tissue without direct injury. A female patient had severe buttock pain and posterior leg numbness after she underwent left-side CPO. Postoperative magnetic resonance imaging showed that the sciatic nerve was strangulated by the surrounding soft tissue. There was no bone fragment, active infection, bone necrosis, tumor, or spine disease. Therefore, we diagnosed nerve palsy by soft tissue strangulation, and revision surgery was indicated. During revision surgery, the sciatic nerve was observed to be strangulated by the scarring soft tissue, and the nerve had no mobility. After detachment, the pain and numbness disappeared. Direct injury of the sciatic nerve should not be caused by CPO; however, there is a possibility of postoperative sciatic nerve palsy due to the scarring soft tissue. Early diagnosis and appropriate treatment are important for optimal postoperative clinical outcomes.

## 1. Introduction

Curved periacetabular osteotomy (CPO) is a procedure used for the treatment of dysplastic hips. Several studies have reported that acetabular reorientation and improvement of acetabular coverage contribute to the decline of dysplastic hip instability [1, 2]. Satisfying long-term results were reported for the traditional procedure [3]. However, various severe complications such as nerve palsy, necrosis of the femoral head, and delayed union or absence of union have been reported in a few cases [4–6]. In contrast, severe complications are rare after CPO [7]. To our knowledge, there is no published report of postoperative sciatic nerve palsy. In the present study, we describe a case of a patient with postoperative sciatic nerve palsy following CPO due to nerve strangulation by the scarring tissue without direct injury.

## 2. Case Presentation

**Figure 1:**
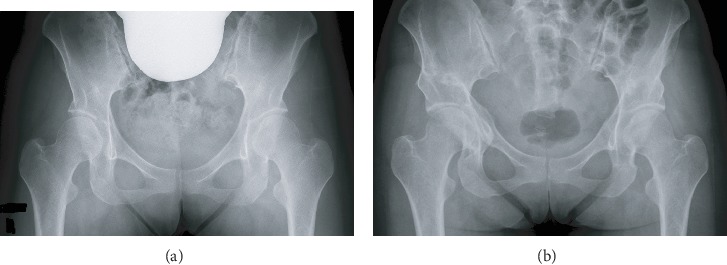
Radiographs. (a) Preoperative pelvic radiograph showing bilateral acetabular dysplasia. (b) Pelvic radiograph after bilateral curved periacetabular osteotomy. At 12 months postoperatively, complete union was observed in both sides.

**Figure 2:**
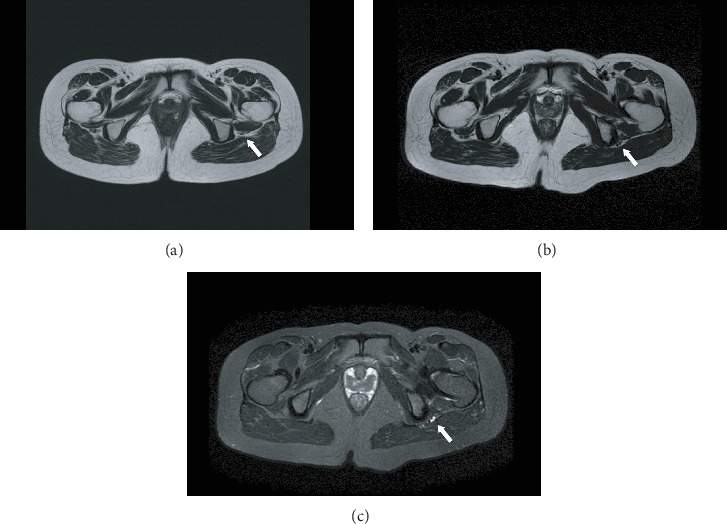
Preoperative and postoperative magnetic resonance imaging. (a) T2-weighted images of preoperative magnetic resonance imaging. The arrow indicates the sciatic nerve and soft tissue without degeneration. (b) T2-weighted images of 12 months postoperative magnetic resonance imaging after left-side curved periacetabular osteotomy. The arrow indicates denatured soft tissue around the sciatic nerve. (c) Short T1 inversion recovery images of 12 months postoperative magnetic resonance imaging after left-side curved periacetabular osteotomy. The arrow indicates intensity change in the sciatic nerve, suggesting nerve degeneration.

A 27-year-old female patient was diagnosed with bilateral dysplastic hips in 2015 in our institution. She had severe pain in the hips and corresponding trochanteric areas after prolonged walking, with a conspicuous decrease in physical activity. Radiography showed bilateral hip dysplasia with early-stage osteoarthritis (Figure 1(a)). Preoperative magnetic resonance imaging (MRI) did not show evidence of any complications such as active infection, bone necrosis, or nerve stenosis. Normal soft tissues around the sciatic nerve area were recognized (Figure 2(a)). Finally, she underwent right- and left-side CPOs in 2016 and 20 months later, respectively. She did not have a past medical history, trauma history, and psychosocial history. There is no family history of coagulation abnormalities. Her coagulation profile was within normal limits, with an activated partial thromboplastin time (APTT) of 31.4 s and a prothrombin time international normalized ratio (PT-INR) value of 0.97.

The patient underwent CPO according to a procedure described in a previous report [8]. Three-dimensional (3D) planning with a 100 mm radius sphere using navigation software (OrthoMap 3D Navigation System; Stryker Orthopedics, Mahwah, NJ, USA) was used for preoperative planning and intraoperative alignment management. The patient was placed on a radiolucent table in the supine position for a direct anterior approach. A skin incision of 9 cm was used for surgical exposure. Before osteotomy, the pelvis was registered with a surface-matching technique [9]. A pubic osteotomy was performed just medial to the iliopubic eminence. A C-shaped osteotomy was performed from the anterior inferior iliac spine to the distal part of the quadrilateral surface along the spherical position and direction. After spherical osteotomy, the acetabular fragment was rotated laterally and anteriorly according to the preoperative planning. After temporal fixation with a Kirschner wire, two or three poly-L-lactic acid screws were used to fix the reoriented acetabular fragment. The surgeries on both sides were performed within a 20-month interval. The same procedure was performed for both sides.

Postoperative radiography showed improvement of the acetabular coverage of the femoral head (Figure 1(b)). The CE angle changed from 22.8° to 35.6° and 20.5° to 31.8° in the right and left sides, respectively. Likewise, the sharp angle changed from 44.2° to 33.5° and 48.9° to 39.1° in the right and left sides, respectively. Partial and full weight bearing were allowed 2 and 12 weeks postoperatively, respectively. Bone union was accomplished uneventfully in both sides. The metal screw was removed 1 year after the CPO. Hip pain disappeared following the right CPO. However, the patient started to feel buttock pain and posterior leg numbness 2 months after the left-side CPO. Indeed, the buttock pain and numbness gradually increased despite conservative treatments such as rehabilitation, nerve block, and pain analgesic administration. Tinel's sign was recognized, and the Straight Leg Raising Test and Bragard's Test results were positive 10 months postoperatively. There was no range of motion limitation or decline in the Manual Muscle Test results of the lower limb. The postoperative MRI 1 year later showed that the sciatic nerve was strangulated by the surrounding soft tissue (Figure 2(b)) and denatured (Figure 2(c)). The increased T2 signal shown in Figure 2(c) was similar to that of the MRI signal of the sutured nerve in a previous study [10]. There was no bone fragment, active infection, bone necrosis, or tumor. In addition, there was no abnormal finding on spine MRI. Therefore, we diagnosed the patient with nerve palsy by soft tissue strangulation, and revision surgery was indicated.

**Figure 3:**
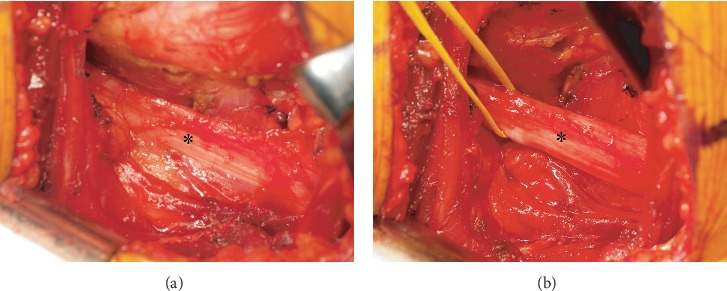
Intraoperative photographs. (a) In the view through the posterolateral approach, the sciatic nerve (^∗^) was strangulated by the scarring soft tissue, and the nerve had no mobility and further extension in hip flexion position. (b) After detachment, the sciatic nerve (^∗^) achieved sufficient mobility.

Revision was performed in the lateral position with a posterolateral approach 2 years postoperatively. After detachment and retraction of the gluteus maximus and external rotator muscles, the sciatic nerve was visualized. The sciatic nerve adhered to and was severely strangulated by the surrounding soft tissue (Figure 3(a)). The nerve was flattened and presented no mobility; further extension of the nerve was performed in the hip flexion position. Then, the nerve was detached from soft tissues, which achieved complete release and adequate mobility of the sciatic nerve (Figure 3(b)). The pain decreased immediately postoperatively. At 1 month after the operation, pain and numbness had disappeared.

## 3. Discussion

Here, we described for the first time, to our knowledge, a case of sciatic nerve palsy following CPO. CPO was developed in 1995 and performed for symptomatic acetabular dysplasia treatment in young adults [1]. Previous reports have shown satisfying clinical outcomes due to reoriented acetabulum, resulting in the improvement of the femoral head coverage and abductor muscle strength [7, 11].

The rate of severe complications following CPO was reported to be very low and related to traditional periacetabular osteotomy [7]. Leunig et al. reported a case of sciatic nerve palsy following periacetabular osteotomy [12]. However, they used Bernese periacetabular osteotomy, and the sciatic nerve palsy was caused by a bone fracture of the posterior column. In our case, the posterior column was intact after surgery. Previous studies have reported that sciatic nerve palsy could be caused by spine diseases, osteophytes, and tumors [13–15]; however, there was no evidence suggesting such causes in this case. The sciatic nerve palsy observed here seemed to have been caused by soft tissue adhesion and strangulation.

Adhesion of soft tissue by surrounding hematoma has been previously reported [16–18]. Hematoma is composed of various cellular, molecular, and induced growth factors, as well as inflammatory cytokines [19]. Hematoma leads to the fibrosis of the surrounding soft tissue [20, 21]. In our case, it is possible that postoperative hematoma from the osteotomy site contributed to the formation of soft tissue scarring and nerve strangulation.

From an intraoperative perspective, the sciatic nerve adhered to and was severely strangulated by the scarring soft tissue, and the detachment of the nerve resulted in mobility of the sciatic nerve and improvement of clinical pain. Pain relief should not be provided without surgical detachment of the nerve. We believe that early diagnosis and treatment are important for the improvement of postoperative clinical outcomes.

In conclusion, we managed a rare case of sciatic nerve palsy following CPO and accomplished pain relief by surgical nerve detachment. Direct injury of the sciatic nerve should not be caused while performing CPO. However, there is a possibility of postoperative sciatic nerve palsy due to soft tissue scarring. Early diagnosis and appropriate treatment are important for optimal postoperative clinical outcomes.
